# No signs of check-list fatigue – introducing the StOP? intra-operative briefing enhances the quality of an established pre-operative briefing in a pre-post intervention study

**DOI:** 10.3389/fpsyg.2023.1195024

**Published:** 2023-06-29

**Authors:** Eliane Timm-Holzer, Franziska Tschan, Sandra Keller, Norbert K. Semmer, Jasmin Zimmermann, Simon A. Huber, Martin Hübner, Daniel Candinas, Nicolas Demartines, Markus Weber, Guido Beldi

**Affiliations:** ^1^Institute for Work and Organizational Psychology, University of Neuchâtel, Neuchâtel, Switzerland; ^2^Department of Visceral Surgery and Medicine, Berne University Hospital, University of Berne, Berne, Switzerland; ^3^Department of Psychology, University of Berne, Berne, Switzerland; ^4^Department of Visceral Surgery, University Hospital Lausanne (CHUV), Lausanne, Switzerland; ^5^Department of Surgery, Triemli Hospital, Zurich, Switzerland

**Keywords:** teamwork in surgery, surgical checklist, intraoperative briefing, patient safety, teamwork in medicine, team intervention

## Abstract

**Background:**

The team timeout (TTO) is a safety checklist to be performed by the surgical team prior to incision. Exchange of critical information is, however, important not only before but also during an operation and members of surgical teams frequently feel insufficiently informed by the operating surgeon about the ongoing procedure. To improve the exchange of critical information during surgery, the StOP?-protocol was developed: At appropriate moments during the procedure, the leading surgeon briefly interrupts the operation and informs the team about the current Status (St) and next steps/objectives (O) of the operation, as well as possible Problems (P), and encourages questions of other team members (?). The StOP?-protocol draws attention to the team. Anticipating the occurrence of StOP?-protocols may support awareness of team processes and quality issues from the beginning and thus support other interventions such as the TTO; however, it also may signal an additional demand and contribute to a phenomenon akin to “checklist fatigue.” We investigated if, and how, the introduction of the StOP?-protocol influenced TTO quality.

**Methods:**

This was a prospective intervention study employing a pre-post design. In the visceral surgical departments of two university hospitals and one urban hospital the quality of 356 timeouts (out of 371 included operation) was assessed by external observers before (154) and after (202) the introduction of the StOP?-briefing. Timeout quality was rated in terms of timeout completeness (number of checklist items mentioned) and timeout quality (engagement, pace, social atmosphere, noise).

**Results:**

As compared to the baseline, after the implementation of the StOP?-protocol, observed timeouts had higher completeness ratings (*F* = 8.69, *p* = 0.003) and were rated by observers as higher in engagement (*F* = 13.48, *p* < 0.001), less rushed (*F* = 14.85, *p* < 0.001), in a better social atmosphere (*F* = 5.83, *p* < 0.016) and less noisy (*F* = 5.35, *p* < 0.022).

**Conclusion:**

Aspects of TTO are affected by the anticipation of StOP?-protocols. However, rather than harming the timeout goals by inducing “checklist fatigue,” it increases completeness and quality of the team timeout.

## 1. Introduction

Besides technical and medical proficiency, teamwork and communication within surgical teams have been identified as crucial factors that impact the surgical process and outcomes (Mazzocco et al., 2009; Sun et al., 2018; Paterson-Brown et al., 2019). In operation rooms (OR), establishing good teamwork is particularly challenging: During surgery, professionals with complementary roles must collaborate. At the operating table, two or more surgeons have to cooperate very closely with each other and with the scrub technician who provides instruments. Anesthesia providers ensure that the patient remains under anesthesia and stable; they often work in parallel with the surgeons, sometimes having to synchronize very closely with them. Circulators are responsible for taking and bringing instruments to the operating table, while also performing administrative duties in parallel with the operation. Because of the different tasks, roles, and perspectives of the team members during an operation, maintaining a shared mental model and high situation awareness may be difficult (Graafland et al., 2015; Afkari et al., 2016). Other challenges to good teamwork in the OR are the notoriously high noise levels which may hamper communication (Keller et al., 2016; Leitsmann et al., 2021), low team familiarity (Kurmann et al., 2014; Stucky et al., 2021) and strong hierarchies, which may hamper psychological safety and diminish speaking up (Appelbaum et al., 2020).

Therefore, interventions have been introduced that aim at fostering better teamwork and communication in the OR (McCulloch et al., 2017; Sun et al., 2018). The best known and nowadays routinely followed intervention is the team-timeout which is part of the WHO surgical safety checklists. The team timeout (TTO) is performed before the operative procedure starts. It has the objective to ensure that OR team members are on the same page about the procedure to be performed and contains checklist items to confirm important information (Haynes et al., 2009). In addition to the team-timeout, other team-related interventions may be employed (McCulloch et al., 2017), such as CRM training, Sun et al. (2018), other checklists (Lyons and Popejoy, 2014), or the StOP?-protocol intraoperative briefing (Tschan et al., 2022) used in the present study.

If multiple interventions are combined or an intervention is added to an existing practice, an important question is whether interventions influence each other. Although there are indications that different team-related interventions may be favorably combined (Buljac-Samardzic et al., 2010; McCulloch et al., 2017) or positively influence one another (Okhuysen and Eisenhardt, 2002), interferences between interventions may also be possible. An example is the tendency to become complacent or even opposed to the use of multiple checklists or interventions, described as “checklist fatigue” (Grigg, 2015).

However, it has rarely been investigated empirically if, and how, interventions influence each other (Buljac-Samardzic et al., 2010). In this prospective observational study using a pre-post design, we evaluate the impact of the introduction of an intraoperative briefing (the StOP?-protocol) on the quality of an already existing briefing (the team-timeout) in surgical departments of three different hospitals.

### 1.1. The team timeout checklist intervention

In 2008, the World Health Organization (WHO) recommended checklist-based team briefings as a standard for surgical teams worldwide (Haynes et al., 2009). These briefings aim to reduce errors and enhance communication and teamwork. One of the recommended briefings is the team timeout (TTO), conducted at the time the patient is anesthetized and prepared, but just before incision. The minimal standard of the TTO includes presentation of all team members, confirming patient identity, surgical procedure, site of incision, and availability of critical images. Surgeons, anesthesia providers and the nursing team inform about anticipated critical events, and the approximate surgery duration is communicated.

In Switzerland, the TTO is not mandatory by law, but it has been adopted by most hospitals (Mascherek et al., 2013; Fridrich et al., 2022); including in the three hospitals participating in this study. Although the WHO suggests which aspects should be discussed during the TTO, it also recommends that the procedure should be adapted for each hospital, indicating that differences between hospital cultures may be important.

The surgical safety checklist (including the TTO) has been related to improved patient outcomes, Haugen et al. (2019), such as reduced negative events, morbidity, and mortality (Lyons and Popejoy, 2014; Haynes et al., 2017; Abbott et al., 2018), and improved team outcomes, including better coordination and communication (Kearns et al., 2011; Molina et al., 2016). Note that not all studies found positive effects (Urbach et al., 2014; Reames et al., 2015).

However, the effectiveness of the TTO depend on its correct use and quality (van Klei et al., 2012). Studies reported low adherence rate and a reluctant adoption of the procedure, particularly for surgeons (Hurlbert and Garrett, 2009), incomplete TTO execution (van Klei et al., 2012; Fridrich et al., 2022), and inattentiveness during the TTO (Biffl et al., 2015). These are not harmless omissions: If boxes are ticked without paying attention, the risk of error detection failures increases (Cullati et al., 2013), and a false sense of security may develop (Russ S. J. et al., 2015). Thus, active participation and commitment by all team members is crucial (Hicks et al., 2014) and team members should not engage in other tasks during the TTO (Vogts et al., 2011). Furthermore, the TTO may create a sense of time pressure. Although a typical TTO takes less than two minutes, some feel that it is taking too long, and start to rush. This may result in omitting information (Vats et al., 2010; Conley et al., 2011) and create a sense of urgency that may induce tensions. A tense atmosphere during the TTO has been found to lead to dismissive communication later on (Vats et al., 2010) and to impaired collaboration throughout the surgery (Whyte et al., 2008; Cullati et al., 2013).

The importance of completeness and quality of the TTO points to the need to avoid additional burdens that may threaten the quality of the TTO. It is thus important to consider if the StOP?-protocol as an additional intervention influences the quality of the TTO.

### 1.2. The StOP?–Intervention

The TTO focuses on exchanging information to prevents omissions and errors, but it cannot cover all necessary information for the whole operation. More specifically, it cannot deal with specific developments that require adapted actions. Indeed, one of the main complaints of surgical team members is feeling under-informed *during* the operation due to the lack of regular updates from surgeons regarding the progress, specific strategic approaches and intraoperative strategy changes (Wauben et al., 2011). Such task-related information exchange during the operation is important, as more information exchange (Mazzocco et al., 2009) and particularly more case-relevant communication have been associated with better patient outcomes (Tschan et al., 2015).

Surgeons are not simply unwilling to share information during the operation with the team. Performing surgery demands high concentration, particularly on manual aspects of the task, and surgeries can be quite stressful for the surgeon (Yamaguchi and Kanemitsu, 2011). Both aspects can impair communication, and high concentration requirements on manual tasks may prevent the surgeon from focusing on the team’s information needs, which requires a change in attentional focus. Focusing on the team constitutes a task in its own right (Fernandez et al., 2008). Stress can lead to team members losing the team perspective (Driskell et al., 1999). If surgeons do communicate as they go, but without a clear shift in attention, their communication may not be properly perceived by team members remote from the table.

To facilitate intraoperative information flow and regular updates, particularly from the surgeons to the team, we developed the StOP?-protocol. This protocol, led by the responsible surgeon, is an intraoperative briefing aimed at exchanging task-and cooperation-related information (Keller et al., 2022; Tschan et al., 2022). During the operation, the surgeon informs the team about the progress of the operation (**St** = status of the surgery), upcoming steps and goals (**O** = objectives), anticipated difficulties (**P** = problems), and encourages team members to ask questions and share observations (**?** = Questions or remarks). Information about status, objectives and potential problems aim at updating the team, asking for active participation aims at encouraging equal information exchange and speaking up (Edmondson, 2003). The structure of the StOP?-intervention is similar to other briefing interventions (Marks et al., 2000; Makary et al., 2006), except that it occurs during the operation at natural breakpoints between subtasks. Between subtasks, concentration requirements for specific aspects of the task are temporally reduced, and it is easier to switch attention to the team level. Multiple StOP?-briefings can be conducted during an operation; surgeons announce when they intend conducting a StOP?-briefing for a specific operation at the end of the TTO.

Research has shown that introducing the StOP?-protocol has positive effects on patient outcomes; it is related to a reduced mortality rate, fewer unplanned reoperations and fewer prolonged hospital stays (Tschan et al., 2022).

### 1.3. Can one team-intervention influence another?

Numerous patient safety interventions have been implemented in surgery over the years, often as a combination of interventions (McCulloch et al., 2017; Storesund et al., 2020).

Both inhibiting and enhancing influences or interferences between different interventions seem possible. For example, adding several checklists may lead to a sense of overregulation (Grigg, 2015) and loss auf of autonomy and even the feeling of infantilization, particularly if checklists are not perceived as well-suited to specific procedures (Grigg, 2015; Dekker, 2018). If checklists multiply, they may be perceived as a hindrance to timely and efficient work (Hales and Pronovost, 2006). If interventions target similar outcomes (as for the TTO and StOP?), people may perceive redundancy (Fourcade et al., 2012). This can create a negative attitude, and medical professionals may develop “checklist fatigue” (Hales and Pronovost, 2006; Grigg, 2015). This may lead to disengagement and reduced adherence (Stock and Sundt, 2015). It is thus possible that anticipating the StoP?-briefing induces aversion and reduces TTO quality.

However, interventions may also positively influence each other. The StOP?-protocol, for instance, builds on and complements the information provided by the TTO during the operation. This may render the information communicated during the TTO more meaningful and useful for the team. Another type of enhancement may be that the introduction of the StOP?-protocol draws attention to team cooperation. In a laboratory setting, Okhuysen and Eisenhardt (2002) explored how simple interventions to foster cooperation improved knowledge integration in groups. One interesting finding of their study was that each of three different interventions not only increased the specifically instructed behavior but spilled over to increase the use of cooperative strategies that were not explicitly instructed. The authors concluded that even simple interventions influence cooperation, as they direct the attention to the team-level and create “windows of opportunity” to switch attention from the task to the team level improving cooperative strategies. Indeed, one study found that teamwork interventions (as compared to system interventions) improved TTO checklist performance (McCulloch et al., 2017). Thus, the introduction of the StOP?-protocol may constitute such a window of opportunity, direct attention to the team process, and thus improve TTO quality. Finally, the introduction of single or combined interventions has been shown to positively influence safety attitudes and the safety climate, which may in turn improve the quality of safety measures (Haynes et al., 2011).

### 1.4. Research questions

Because both negative and positive effects of the introduction of a new briefing on an existing intervention are plausible, we do not formulate directed research questions.

The first research question thus was to compare the completeness and the quality of the TTO, as assessed by trained observers, before and after the StOP?-protocol was introduced, to assess potential effects of the additional intervention on the TTO.

A secondary research question was to evaluate differences between participating hospitals in completeness and quality of TTO as well as in the effect of the StOP? intervention on the TTO.

## 2. Methods

### 2.1. Sample

The study was conducted in the general surgery departments of two large Swiss University Hospitals and in the general and vascular department of a middle-sized urban hospital. These hospitals agreed to participate in a larger study that aimed to investigate the effects of the StOP?-protocol on patient outcomes, using a before-after design and comparing a nine-month baseline with nine-month intervention period (Tschan et al., 2022).

For this smaller observational study, we strove to assess a mix of elective surgeries from the larger study that was typical for each hospital. Criteria to include operations during the nine-month baseline period were elective general or vascular surgeries with an expected duration of more than 1 hour, and observers had to be available. Exclusion criteria were a preexisting surgical site infection (e.g., re-operation after the patient suffered an infection) or another surgery at the same site within the last 30 days. During the intervention period, case-mix and observer availability were once again limiting factors, but we aimed to match the proportion of the different types of surgery observed during the baseline period. In total, 371 operations were observed; and a TTO was performed in 366 of these operations (98.7%). The sample size was determined by the eligibility criteria, and we did not conduct a post-hoc power analysis in accordance with current recommendations (Dziak et al., 2020). The characteristics of the operations are reported in the result section. Due to the typically unstable composition of surgical teams, which can change even within an operation (Stucky and De Jong, 2021); and to assure confidentiality, we did not collect data on specific team members. All analyses are on the team level.

### 2.2. Measures

#### 2.2.1. Characteristics of operations

Operations performed were coded into 11 different categories as (1) Upper gastrointestinal (GI) tract (e.g., small bowel) (2) Lower GI tract (e.g., hemicolectomy), (3) Liver (e.g., liver resection). (4) Pancreas (e.g., Whipple procedure), (5) Hernia (e.g., inguinal hernia), (6) cholecystectomy, (7) Gastric bypass/sleeve, (8) Kidney transplants, (9) Thoracoscopy (e.g., wedge resection), (10) vascular surgery (e.g., vascular bypass), and (11) other procedures. Data for patient age and gender were collected for each operation.

#### 2.2.2. Intervention, context

It was coded whether the operation took place during the baseline or during the intervention period (0.1). To account for organizational differences, it was coded in which of the three hospitals (A, B, C) the intervention took place, using a dummy code.

#### 2.2.3. Team timeout completeness

The goal of the TTO is to assure that all mandatory checklist items are checked before incision. Team timeout completeness (i.e., discussing each item on the list) therefore is an important quality measure (Cullati et al., 2013; Pickering et al., 2013; Fridrich et al., 2022). TTO completeness indicates whether the items on the checklist are referred to. However, hospitals are encouraged to adapt the TTO checklist to their specific circumstances and needs (Weiser et al., 2010); therefore, the number of items on the checklist, the number of mandatory items to discuss, as well as the specific way of performing the TTO differed across hospitals. In Hospital A, the TTO had eleven items, all of them mandatory. The TTO was initiated and led by the circulating nurse who read out aloud each of the items. Responses were provided by the person responsible for the respective information (e.g., the anesthesiologist for allergies, the surgeon for potential blood loss, the scrub nurse for instruments). In Hospitals B and C, the TTO was initiated by the responsible surgeon and predominantly entailed communication between the surgeon and anesthesiology providers. The TTO checklist of Hospital B had six items, two were mandatory (patient identity and planned procedure); the TTO of Hospital C had six items, three of them mandatory (patient identity, planned procedure, prophylactic antibiotics). In hospital B and C, the non-mandatory items were only mentioned if considered relevant by the surgeon or anesthetists. To assure comparability across hospitals, TTO completeness was calculated as proportion of mandatory items communicated for each hospital. TTO completeness for Hospital A was the proportion of the 11 mandatory items discussed. For Hospital B and C, we calculated two completeness scores; one related to the mandatory items (B: 0, 0.5 or 1; C: 0, 0.33, 0.66 or 1), and one expressed as proportion of all six items on the list (all items). If the communication during the TTO was not audible enough to determine if an item was mentioned or not, the data was coded as missing; scores were only calculated if there was data for every item. None of the hospitals had established a formal sign-out procedure.

#### 2.2.4. Team timeout quality

The TTO quality was assessed by trained observers (work psychologists) using an adapted version of known TTO quality measures (Vogts et al., 2011; Fourcade et al., 2012; Levy et al., 2012; Pickering et al., 2013; Russ S. et al., 2015). In addition to contextual aspects of the TTO (e.g., who was present, who initiated it), which are not reported here, four components of TTO quality were assessed: **Engagement** during TTO was assessed using a 5-point Likert scale ranging from *not committed* (1) to *committed* (5); **Pace** of the TTO was assessed using a 5-point Likert scale ranging from *rushed* (1) to *calm* (5); **Social climate** was assessed using a 5-point Likert scale ranging from *irritated* (1) to *serene* (5); **Noisy conditions** was assessed using a 5-poing Likert scale ranging from *no noise* (1) to *very noisy* (5). The scales provided explicit categories for the extremes, and observers were instructed to indicate the level of agreement based on the numerical values assigned to each option. After reversing the noise item, the quality components were combined into a quality index, which demonstrated good internal consistency (Cronbach’s α =0.697). About 9% (N = 33) of the observed TTO were assessed independently by two observers, and intra class correlation (ICC) was calculated to assess inter-observer agreement, yielding good results (engagement: ICC = 0.741; pace: ICC = 0.818; social climate: ICC = 0.749; noise: ICC = 0.854).

### 2.3. Study design

This was a prospective intervention study employing a pre-post design. The implementation consisted of the introduction of the StOP?-protocol described in the introduction. During the baseline period, the surgical team did not get any instruction related to their behavior or communication. To prepare the intervention, surgeons were individually trained on how and when to perform the StOP?-protocol. Scrub technicians and circulators as well as anesthesia providers were also informed about the StOP?-protocol.

Observer-based assessment of TTO completeness and quality during the baseline period (9 months) before the implementation of the StOP?-protocol was compared with observations during the intervention period. All TTO were observed *in vivo* by observers present in the OR. Surgical team members were aware of the presence of observers, but neither the members of the surgical team nor the members of the observational team were aware of the specific research question.

The study was conducted in accordance with the principles outlined in the Helsinki protocol for human subject research and was approved by the ethics committees (leading committee #161/2014). Consent from the team members to be observed was based on an opt-out procedure; teams were asked for permission to be observed before the operation, and each member of the team could at any moment before and during the process ask the observers to leave. Patient consent for two hospitals was based on general consent; in one hospital, the local ethical committee also approved inclusion of operations for patients who did not refuse the use of their data.

### 2.4. Statistics

Descriptive statistics are reported as means and standard deviations, or counts and percentages for categorical variables. To compare TTO quality before and after the intervention across the hospitals, we conducted 2×3 factorial ANOVA’s, with the StOP?-intervention (before, after) and the hospital (Hospital A, Hospital B, Hospital C) as fixed factors. Pairwise comparisons (before and after the intervention and between the hospitals) were assessed based on estimated marginal means and were Bonferroni adjusted; differences between hospitals in the rate of change were assessed by an intervention x hospital interaction effect; effect sizes are partial eta squared. Interobserver reliability was assessed by intraclass correlation (ICC). P less than 0.05 was considered statistically significant. We used SPSS 28 for all analyses (IBM, 2021).

## 3. Results

### 3.1. Characteristics of operations

A total of 371 operations were observed. Table 1 shows the mix of operations observed during the baseline and intervention period for each hospital. Comparing the proportion of surgery types observed before and after the intervention yielded no significant differences, indicating successful matching.

**Table 1:** Operations observed during baseline and Intervention per hospital.

		Hospital A		Hospital B		Hospital C	
		Baseline	Intervention	Baseline	Intervention	Baseline	Intervention
*N*		76	75	43	77	46	54
Patient Age		58.41	58.55	56.02	62.32	64.66	61.58
Sex	Male (56.6%)	43	49	25	41	25	27
	Female (43.4%)	33	26	18	36	21	27
Type of surgery							
	Upper GI tract	7	8	4	7	2	2
	Lower GI tract	11	12	9	16	5	11
	Liver	16	13	7	11	1	2
	Pancreas	16	14	7	10	3	3
	Hernia	4	4	1	7	12	11
	Cholecystectomy	4	4	4	12	7	8
	Gastric bypass/sleeve	6	5	6	6	4	4
	Kidney transplants	8	8		1		
	Thoracoscopic					5	6
	Vascular surgery					4	6
	Other	4	7	5	7	3	1
Chi^2^			1.46 (df = 8, *p* = 0.99)		4.78 (df = 8, *p* = 0.78)		3.57 (df = 9, *p* = 0.94)

Chi^2^ statistics refer to the difference between surgical type during baseline and intervention period, per hospital.

### 3.2. Team timeout completeness

In 356 of the 366 operations with observed TTO, completeness of the time-out procedure could be assessed. Descriptive statistics and ANOVA results of TTO completeness are displayed in Table 2. Our analysis focuses on the mandatory items of the checklist; for results concerning all items (which were very similar), see Supplementary Table S1. Analyses showed a positive effect of the StOP? intervention on TTO completeness (Table 2, line “Intervention”). Regarding hospitals, TTO completeness was significantly higher in Hospital A than in Hospitals B and C. Completeness was somewhat higher in Hospital B as compared to Hospital C, but that difference was not significant. These results indicate that the introduction of the StOP?-protocol did have positive effects on the completeness of the TTO. There was no significant interaction effect (intervention × hospital).

**Table 2:** Timeout completeness before and after the StOP?–Intervention and between hospitals: mandatory items.

TTO completeness (mandatory items)											
	Total		Baseline		Intervention						
	*N*	*M* (SD)	*N*	*M* (SD)	*N*	*M* (SD)	Difference** intervention –baseline (SE)	95% CI for difference	*F*	*P*	Partial eta squared
Model									6.75	<0.001	
Intervention	356	0.95 (0.14)	154	0.94 (0.16)	202	0.97 (0.12)	0.05 (0.02)	0.002 to 0.08	8.69	0.003	0.024
											
Hospital A	149	0.99 (0.04)	76	0.99 (0.51)	73	1.00 (0.10)					
Hospital B	116	0.94 (0.20)	39	0.91 (0.25)	77	0.96 (0.16)					
Hospital C	91	0.90 (0.16)	39	0.86 (0.17)	52	0.94 (0.15)					
							Difference** between Hospitals	95% CI for difference	*F*	*P*	
Between Hospitals									13.62	<0.001	0.072
Hospital A–B							0.06 (0.02)	0.02 to 0.1			
Hospital A–C							0.09 (0.02)	0.05 to 0.14			
Hospital B–C							0.04 (0.02)	−0.01 to 0.08			
Intervention × Hospital									1.47	0.232	0.008

*Completeness scores are shown as proportions. ** Based on estimated marginal means.

### 3.3. Team timeout quality

Descriptive statistics and ANOVA results for the TTO quality index and for each of the components of the quality index are displayed in Tables 3, 4. Analyses show a significant positive relation between the StOP? intervention and the TTO quality index (Table 3), line “Intervention”), but also for each component separately (Table 4), line “Intervention,” indicating that engagement, pace, and social climate during the TTO improved during the StOP? intervention, whereas noise during TTO decreased. Regarding the secondary research question, the analyses showed that TTO quality in Hospital A was significantly higher than in Hospital B before, but also during the intervention, both for the quality index and for the quality components (line “between hospitals” in Tables 3, 4). For Hospital C, the intervention had no significant effects on the quality index nor on the components engagement, pace and noise, and the component social climate in Hospital C was actually significantly lower after the intervention; the interaction hospital x intervention was significant for the quality index and the components engagement, social climate, and noise, but not for pace of the TTO, indicating that the intervention had differential effects in different hospitals.

**Table 3:** Quality index TTO before and after the StOP?-intervention and between hospitals.

Quality index TTO*											
	Total		Baseline		Intervention						
	*N*	*M* (SD)	*N*	*M* (SD)	*N*	*M* (SD)	Difference** intervention –baseline (SE)	95% CI for difference	*F*	*P*	Partial eta squared
Model									31.87	<0.001	
Intervention	366	4.03(0.72)	162	3.90(0.75)	204	4.12(0.68)	0.30 (0.07)	0.17 to 0.43	21.53	<0.001	0.056
											
Hospital A	149	4.25 (0.59)	76	4.08 (0.59)	73	4.43 (0.54)					
Hospital B	118	3.53 (0.77)	41	3.13 (0.68)	77	3.53 (0.76)					
Hospital C	99	4.28 (0.49)	45	4.31 (0.54)	54	4.26 (0.45)					
							Difference** between Hospitals	95% CI for difference	F	P	
Between Hospitals									71.25	<0.001	0.284
Hospital A–B							0.82 (0.08)	0.64 to 1.01			
Hospital A–C							−0.03 (0.08)	−0.22 to 0.16			
Hospital B–C							−0.85 (0.08)	−1.05 to-0.65			
Intervention × Hospital									7.47	0.001	0.040

*The quality index is the mean of engagement, pace, social atmosphere and (reversed) noise, range from 1 to 5. **Based on estimated marginal means.

**Table 4:** Quality of TTO for the quality components engagement, pace, social climate and noise before and after the StOP?–intervention and between Hospitals.

Engagement during TTO											
	Total		Baseline		Intervention						
	*N*	*M* (SD)	*N*	*M* (SD)	*N*	*M* (SD)	Difference* intervention –baseline (SE)	95% CI for difference	*F*	*p*	Partial eta squared
Model									17.22	<0.001	
Intervention	366	3.93 (0.97)	162	3.78 (1.01)	204	4.04 (0.93)	0.35 (0.10)	0.16–0.54	13.48	<0.001	0.036
											
Hospital A	149	4.14 (0.74)	76	3.95 (0.73)	73	4.34 (0.63)					
Hospital B	118	3.39 (1.15)	41	3.00 (1.18)	77	3.60 (1.08)					
Hospital C	99	4.25 (0.79)	45	4.22 (0.88)	54	4.28 (0.71)					
							Difference* between Hospitals	95% CI for difference	F	P	
Between hospitals									38.36	<0.001	0.176
Hospital A–B							0.85 (0.11)	0.58 to 1.12			
Hospital A–C							−0.11 (0.12)	−0.38 to 0.17			
Hospital B–C							−0.95 (0.12)	−1.25 to −0.66			
Intervention × Hospital									2.47	0.09	0.014

Pace of TTO											
	Total		Baseline		Intervention						
	*N*	*M* (SD)	*N*	*M* (SD)	*N*	*M* (SD)	Difference* intervention –baseline (SE)	95% CI for difference	*F*	*p*	Partial eta squared
Model									8.93	<0.001	
Intervention	366	3.84 (1.12)	162	3.64 (1.18)	204	4.00 (1.05)	0.44 (0.12)	0.22 to 0.67	14.85	<0.001	0.040
											
Hospital A	149	4.07 (1.01)	76	3.87 (1.06)	73	4.29 (0.92)					
Hospital B	118	3.43 (1.14)	41	2.98 (1.17)	77	3.68 (1.15)					
Hospital C	99	3.98 (1.04)	45	3.87 (1.16)	54	4.07(0.93)					
							Difference* between Hospitals	95% CI for difference	*F*	*p*	
Between hospitals									16.97	<0.001	0.086
Hospital A–B							0.75 (0.14)	0.43 to 1.08			
Hospital A–C							0.11 (0.14)	−0.23 to 0.44			
Hospital B–C							−0.65 (0.15)	−1.00 to −0.29			
Intervention × Hospital									1.39	0.25	0.008

Social climate TTO											
	Total		Baseline		Intervention						
	*N*	*M* (SD)	*N*	*M* (SD)	*N*	*M* (SD)	Difference* intervention –baseline (SE)	95% CI for difference	*F*	*p*	Partial eta squared
Model									9.03	<0.001	
Intervention	366	4.31 (0.80)	162	4.22 (0.82)	204	4.40 (0.77)	0.20 (0.08)	0.04 to 0.36	5.83	0.016	0.016
											
Hospital A	149	4.35 (0.80)	76	4.21 (0.81)	73	4.49 (0.77)					
Hospital B	118	4.07 (0.88)	41	4.03 (0.84)	77	4.37 (0.82)					
Hospital C	99	4.60 (0.58)	45	4.70 (0.51)	54	4.44 (0.60)					
							Difference* between Hospitals	95% CI for difference	*F*	*p*	0.081
Between hospitals									15.88	<0.001	
Hospital A– B							0.37 (0.10)	0.14 to 0.60			
Hospital A–C							−0.22 (0.10)	−0.45 to 0.02			
Hospital B–C							−0.58 (0.11)	−0.84 to −0.33			
Intervention × Hospital									7.37	0.001	0.039

Noise** during TTO											
	Total		Baseline		Intervention						
	*N*	*M* (SD)	*N*	*M* (SD)	*N*	*M* (SD)	Difference * intervention –baseline (SE)	95% CI for difference	*F*	*p*	Partial eta squared
Model									32.47	<0.001	
Intervention	366	1.98 (1.10)	162	2.03 (1.08)	204	1.95 (1.03)	−0.22 (0.10)	−0.41 to −0.03	5.32	0.022	0.015
											
Hospital A	149	1.56 (0.78)	76	1.71 (0.88)	73	1.40 (0.64)					
Hospital B	118	2.79 (1.11)	41	3.15 (0.99)	77	2.60 (1.13)					
Hospital C	99	1.67 (0.77)	45	1.56 (0.73)	54	1.76 (0.80)					
							Difference*between Hospitals	95% CI for difference	*F*	*p*	
Between Hospitals									78.75	<0.001	0.304
Hospital A–B							−1.32 (0.11)	−1.59 to −1.05			
Hospital A–C							−0.10 (0.12)	−0.38 to 0.17			
Hospital B–C							1.21 (0.12)	0.92 to 1.51			
Intervention × Hospital									4.88	0.008	0.026

*Based on estimated marginal means. **less noise indicates better quality.

## 4. Discussion

The introduction of the StOP?-protocol in surgical wards was associated with the improvement in the quality of the TTO. These improvements encompassed completeness, engagement, pace, social climate, and noise conditions. Thus, the additional briefing did not have a negative effect on the already established briefing; rather, the intervention was related to a better TTO quality. Even in the hospital where the TTO did not improve following the intervention, only one component, social climate, declined significantly; the other components, did not change significantly.

These results are consistent with the findings by Okhuysen and Eisenhardt (2002) in a different field, as well as with previous research investigating the effects of team training interventions on TTO quality (McCulloch et al., 2017). One possible explanation for this effect is that an additional briefing opens the opportunity for teams to focus their attention on the team level. This may positively influence cooperative behavior beyond the specific target of the intervention. The effect could be due to momentary effects, whereby the anticipation of the StOP?-briefing enhances the overall attention of the team. However, it could also be a more general effect, resulting from the information and training provided for the StOP? intervention, as well as the regular refresher training. These activities may have served as reminders to team members about the importance of information exchange and collaboration in the OR.

There were marked differences in TTO quality between the hospitals, as well as some significant interaction effects, indicating differences in the impact of the intervention across hospitals. Notably, although there was an overall positive association between the StOP?-protocol and TTO quality, introducing the StOP?-protocol did not influence the quality of the TTO index or its components engagement, pace, and noise conditions in Hospital C. This lack of impact may be due to a ceiling effect, as the values in Hospital C were already close to the scale maximum before the intervention and were higher compared to the other hospitals, leaving limited room for improvement. However, the social climate during the TTO in Hospital C was significantly lower after the introduction of the StOP?. Again, this outcome may be explained by a ceiling effect or a regression toward the mean effect. Note that the social climate score before intervention was 4.7 (on a scale from 1 to 5) which decreased to 4.44 after the intervention. Social climate was markedly higher in Hospital C than in the other hospitals before the intervention but was similar and still high after the intervention. Nevertheless, alternative explanations cannot be ruled out.

When comparing hospitals, the overall TTO quality in Hospital B was lower than in Hospital A, both before and after the introduction of the StOP?-protocol. In general, hospital effects were larger than the effects of the intervention, as indicated by the partial eta squared measure. This finding confirms the presence of cultural differences between hospitals, a well-established fact (Sexton et al., 2006; Körner et al., 2015).

There was concern regarding the potential of negative effects of the StOP-protocol on the TTO, because it could lead to perceived redundancy and checklist fatigue (Hales and Pronovost, 2006; Grigg, 2015). In healthcare, some level of redundancy is generally favored as it enhances safety by reducing the risk of errors with multiple checks by different persons (Sivathasan et al., 2010). However, too much redundancy can also lead people to skip information checking, as they feel the information was already checked enough (Fourcade et al., 2012; Papaconstantinou et al., 2013). That the StOP?-intervention evidently did not lead to perceived inappropriate redundancy during the TTO and did not negatively impact the TTO quality suggests that the addition of a single briefing was not enough to induce a sense of overload. Moreover, note that the StOP?-protocol addresses other kinds of information than the TTO. Therefore, it may not be perceived as “just another checklist,” but rather as the exchange of task-and cooperation-relevant information pertaining to the procedure and to strategic changes. This argument is supported by the positive effects of the StOP?-protocol on patient outcomes (Tschan et al., 2022), and team outcomes, such as perceived collaboration quality, situation awareness, and ease of speaking up (Tschan et al., Submitted). Additionally, the StOP? protocol is not time-consuming to perform and easy to follow, and it facilitates communication among the members of the team.

This study has several limitations. Firstly, the sample size is relatively low, as only surgeries could be included for which observers were available which may also limit the representativeness of the surgeries performed. In addition, all participating surgical departments are located in midsize and large hospitals and predominantly specialize in general (visceral) and vascular surgery, thus limiting the generalizability of the findings to other surgical specialties and smaller settings.

Another limitation is that random assignment was not feasible for this intervention, so a pre-post design had to be employed. Furthermore, participants and observers were aware of the intervention, as this could not be blinded. However, neither the surgical teams nor the observers were aware of the specific research question investigated in this paper, mitigating some potential biases.

Also, we cannot entirely exclude the possibility that an item was not registered despite being mentioned in the TTO because the observer simply did not hear (or understand) it. But even if we account for this possibility, the increased TTO completeness remains noteworthy. Furthermore, the TTO should be executed loud enough to be audible for the whole OR, even for someone at the other side of the room. Lastly, like in any observational study, there is the limitation that other unmeasured factors or variables could have influenced the results.

This study has practical implications, demonstrating that the already established TTO procedure benefited from another briefing intervention overall in two out of the three hospitals. In addition, even in the hospital that did not show improvement, results did not indicate an effect akin to “checklist fatigue” or a negative impact on the TTO. While the TTO has been recognized for its positive effects on team collaboration (Lingard et al., 2008), its scope and purpose are limited. This study demonstrates that an additional intervention fostering information exchange during the operation can be beneficial and even improve the quality of an already established briefing. However, it is crucial to note that the effectiveness of each additional intervention cannot be assumed and needs to be investigated individually.

## Data availability statement

The datasets presented in this article are not readily available because the raw data are available upon request from the corresponding author to researchers eligible to work with codified personal health care data under Swiss legislation. Eligibility will be determined by Kantonale Ethikkomission Bern when needed. Requests to access the datasets should be directed to guido.beldi@insel.ch.

## Ethics statement

The studies involving human participants were reviewed and approved by Kantonale Ethikkommission Bern (KEK); KEK-Gesuchs_Nr: 161/14. Written informed consent for participation was not required for this study in accordance with the national legislation and the institutional requirements.

## Author contributions

ET-H, FT, SK, NS, GB, DC, ND, and MW: study conception. ET-H, JZ, and SH: data collection (including conceptual aspects). ET-H and FT: data analysis. ET-H, FT, SK, NS, JZ, MH, MW, DC, ND, and GB: substantial contributions to manuscript. All authors contributed to the article and approved the submitted version.

## Funding

This project was funded by grant CR13I3_156882 from the Swiss National Science Foundations and supported by the Deutsche Bosch-Stiftung, grant # 12.5.A381.0040.0. The funders and supporters of the study had no role in study design, data collection, analyses, interpretation, or writing. Open access funding by University of Neuchâtel.

## Conflict of interest

The authors declare that the research was conducted in the absence of any commercial or financial relationships that could be construed as a potential conflict of interest.

## Publisher’s note

All claims expressed in this article are solely those of the authors and do not necessarily represent those of their affiliated organizations, or those of the publisher, the editors and the reviewers. Any product that may be evaluated in this article, or claim that may be made by its manufacturer, is not guaranteed or endorsed by the publisher.
