# Supplementary material for: No signs of check-list fatigue – introducing the StOP? intra-operative briefing enhances the quality of an established pre-operative briefing in a pre-post intervention study
Source: Front Psychol. 2023 Jun 29;14:1195024. doi: 10.3389/fpsyg.2023.1195024 (PMC10338924; doi:10.3389/fpsyg.2023.1195024)
Supplement: Supplementary file 1 [file Table_1.docx]

Supplementary Material

No signs of check-list fatigue – Introducing the StOP? intra-operative briefing enhances the quality of an established pre-operative briefing: a pre-post intervention study

Timm-Holzer, E.*; Tschan, F.**, Keller, S.; Semmer, N.K; Zimmermann, J., Huber, S.A., Hübner, M., Candinas, D., Demartines, N., Weber, M., & Beldi, G.***

**Correspondence:** Corresponding Authors: [*Eliane.timm@unibe.ch](mailto:*Eliane.timm@unibe.ch); [**Franziska.tschan@unine.ch](mailto:**Franziska.tschan@unine.ch); [***Guido.Beldi@insel.ch](mailto:***Guido.Beldi@insel.ch)

# Supplementary Tables

TABLE S1: **Timeout Completeness** before and after the StOP?-intervention and between hospitals: including mandatory and non-mandatory items

| **Completeness TTO (all items)** | | | | |  | |  |  |  |  |
| --- | --- | --- | --- | --- | --- | --- | --- | --- | --- | --- |
|  | **Total** | | **Baseline** | | **Intervention** | |  |  |  |  |
|  | N | M(SD) | N | M(SD) | N | M(SD) | Difference**  intervention –baseline (SE) | 95% CI for difference | F | P |
| Model |  |  |  |  |  |  |  |  | 57.81 | <0.001 |
| Interven-tion | 334 | .85(.21) | 141 | .83(.23) | 193 | .86(.19) | .089(.02) | .05 to .13 | 23.35 | <0.001 |
|  |  |  |  |  |  |  |  |  |  |  |
| Hospital A | 149 | 0.99(0.04) | 76 | 0.99(0.05) | 73 | 1.00(0.01) |  |  |  |  |
| Hospital B | 112 | 0.78(0.23) | 38 | 0.68(0.24) | 74 | 0.83(0.21) |  |  |  |  |
| Hospital C | 73 | .66(.19) | 27 | .59(.2) | 46 | .7(.16) |  |  |  |  |
|  |  |  |  |  |  |  | Difference** between Hospitals | 95% CI for difference | F | P |
| Between Hospitals | | |  |  |  |  |  |  | 140.35 | <0.001 |
| Hospital A - B | | |  |  |  |  | 0.24(0.02) | 0.20 to 0.27 |  |  |
| Hospital A - C | | |  |  |  |  | .35(.02) | .3 to .41 |  |  |
| Hospital B - C | | |  |  |  |  | .11(.03) | .05 to .17 |  |  |
| Intervention x Hospital | | | |  |  |  |  |  | 5.88 | 0.003 |

**
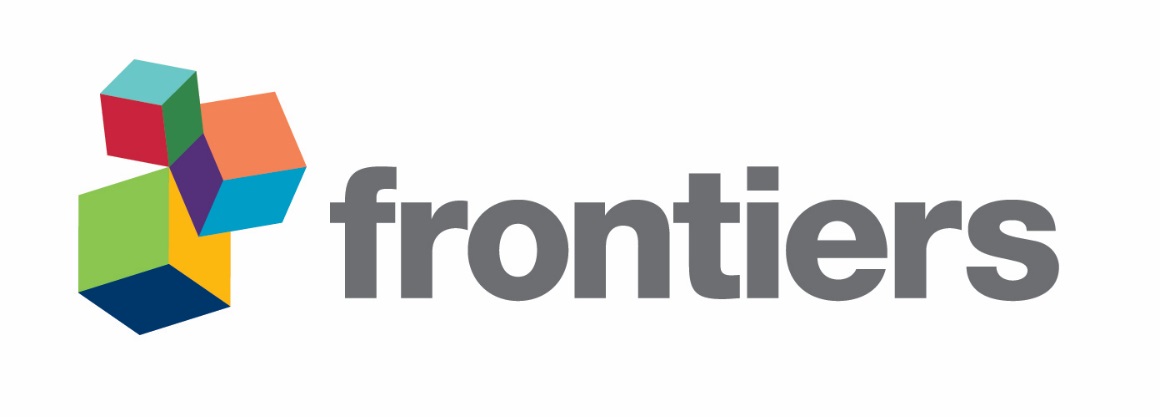
**
